# *Cladolasma ailaoshan*, a new species of the genus *Cladolasma* Suzuki, 1963 from China (Opiliones, Nemastomatidae, Ortholasmatinae)

**DOI:** 10.3897/zookeys.748.23273

**Published:** 2018-04-04

**Authors:** Feng Zhang, Likun Zhao, Chao Zhang

**Affiliations:** 1 The Key Laboratory of Invertebrate Systematics and Application, College of Life Sciences, Hebei University, Baoding, Hebei 071002, China

**Keywords:** Ailao Mountain, *Dendrolasma*, harvestmen, new species, taxonomy

## Abstract

The fourth species of the Asian genus *Cladolasma*, *C. ailaoshan* **sp. n.** is described from specimens collected in Yunnan Province, China. The new species is distinct from *C. parvulum* Suzuki, 1963 and *C. angka* (Schwendinger & Gruber, 1992) in lacking enlarged, dorsally-directed tubercles on the abdominal scutum; and from *C. damingshan* Zhang & Zhang, 2013 in having keels around the eyes and in the position of the eyes. Differences in male genital structures between the Chinese species are small, while there are more differences with the Japanese species.

## Introduction

The genus *Cladolasma* Suzuki, 1963 was reinstated by Shear (2010), and is represented by three species restricted to Asia: China (*C. damingshan* Zhang & Zhang, 2013), Japan (*C. parvulum* Suzuki, 1963), and Thailand (*C. angka* Schwendinger & Gruber, 1992). The representatives of *Cladolasma* are tiny soil- and litter-dwelling harvestmen, usually found at high-altitude areas, e.g., *C. damingshan* at 1231 m, *C. parvulum* at 1200–1500 m, and *C. angka* at 2530 m (Zhang and Zhang 2013; Suzuki 1974; Schwendinger and Gruber 1992).

During biodiversity surveys, intensive collections were made at Ailaoshan National Natural Reserve in August 2011 by the personnel of the Xishuangbanna Tropical Botanical Garden, Chinese Academy of Sciences. Among the collected specimens, a new species, *C. ailaoshan* sp. n. is recognized and described below. This constitutes the second species of the genus recorded from China.

## Materials and methods

Specimens were extracted using Berlese funnels by Akihiro Nakamura at Ailaoshan, Xishuangbanna Tropical Botanical Garden, Chinese Academy of Sciences, China, preserved in 75% ethanol, examined, and drawn under a Leica M205a stereomicroscope equipped with a drawing tube. Morphological terminology mostly follows Gruber (2007), Schwendinger and Gruber (1992), Shear and Gruber (1983), and Suzuki (1974). All measurements follow Shear (2010) and are given in millimeters (mm). Terminology for genital structures follows Shear and Gruber (1983), Martens (1986) and Macías-Ordóñez et al. (2010). Type specimens are deposited in the Museum of Hebei University, Baoding, China (MHBU).

## Taxonomy

### Nemastomatidae Simon, 1872

#### Ortholasmatinae Shear & Gruber, 1983

##### Cladolasma

Taxon classificationAnimaliaOpilionesNemastomatidae

Suzuki, 1963

Cladolasma Suzuki, 1963: 40–41; Shear 2010: 17–18; Zhang and Zhang 2013: 444.
Dendrolasma : Suzuki 1974: 121–122; Shear and Gruber 1983: 51; Schwendinger and Gruber 1992: 57. [Cladolasma was placed in the synonymy of Dendrolasma by Suzuki (1974) and revalidated by Shear (2010)].

###### Type species.

*Cladolasma parvula* Suzuki, 1963, by monotypy and original designation.

###### Distribution.

China (Guangxi, Yunnan), Thailand (Doi Sutep), Japan (Kyushu).

##### Cladolasma ailaoshan sp. n.

Taxon classificationAnimaliaOpilionesNemastomatidae

http://zoobank.org/66D046DE-CEE9-4378-BB85-6A914E82FE36

Figs 1–4 , 5–12 , 13–18 , 19–24

###### Diagnosis.

**Figures 1–4.**
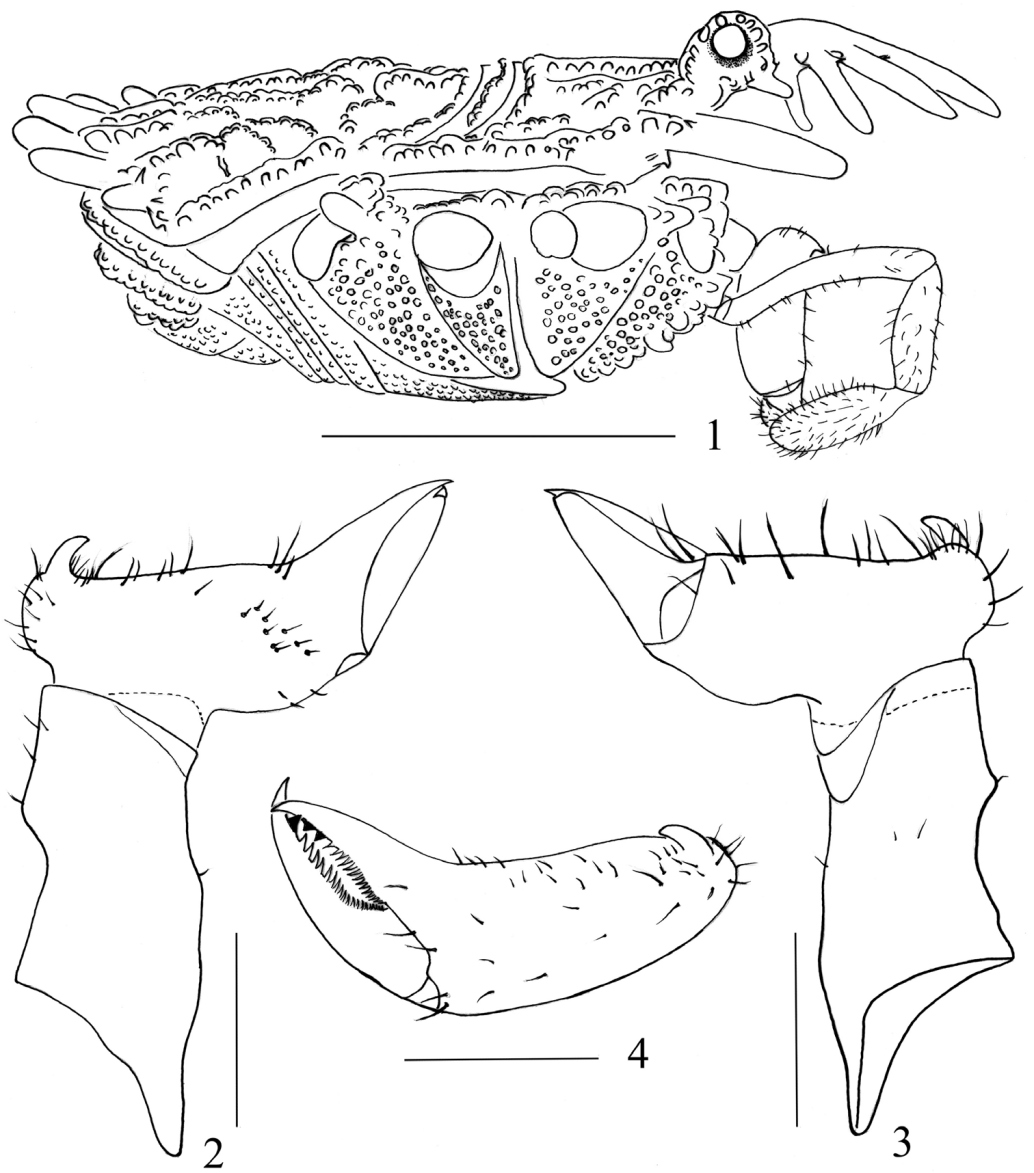
*Cladolasma ailaoshan* sp. n. male (holotype) **1** Body, lateral view **2** Left chelicera, prolateral view **3** Left chelicera, retrolateral view **4** Second segment of chelicera, dorsal view. Scale bars: 1 mm (**1**); 0.25 mm (**2–4**).

**Figures 5–12.**
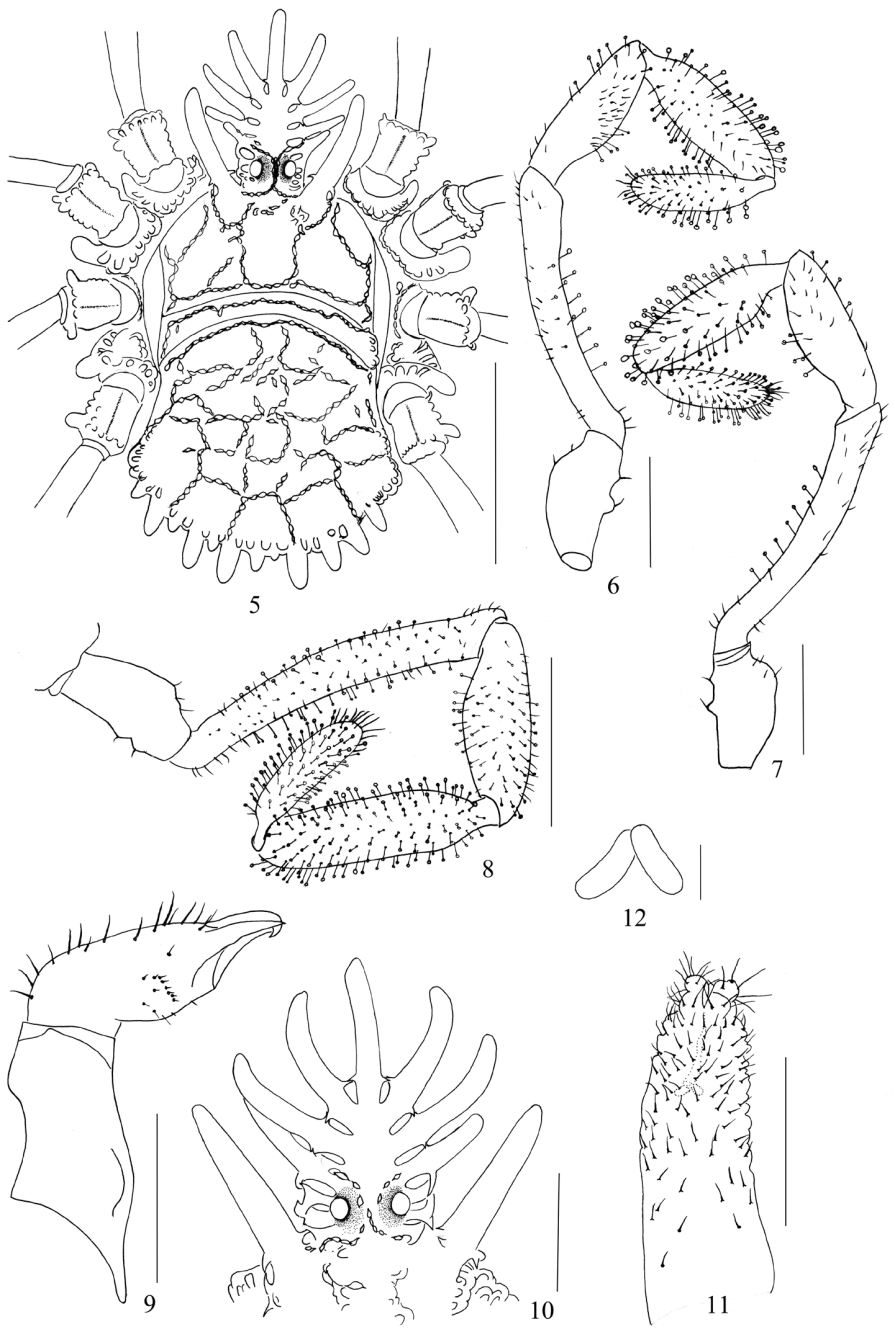
*Cladolasma ailaoshan* sp. n. **5** Body, male, dorsal view **6** Left pedipalp, male, prolateral view **7** Left pedipalp, retrolateral view **8** Left pedipalp, female, prolateral view **9** Left chelicera, female, prolateral view **10** Hood, female, dorsal view **11** Ovipositor **12** Receptacula seminis. Scale bars: 1 mm (**5**); 0.5 mm (**8–11**); 0.25 mm (**6–7**); 0.625 mm (**12**).

Base of penis dorso-ventrally depressed, truncus bent at base (Fig. 14). Glans with 10 small spines and two large lateral spines: six small ones arranged around base of stylus; two small ones situated on dorsal and ventral side separately (Figs 15–18). Ocularium (Figs 1, 5, 10, 19, 21–23) with circumocular keels (see Suzuki 1974: 123, fig. 1 for *C. parvulum*; Schwendinger and Gruber 1992: 58, fig. 2 for *C. angka*; in comparison to Zhang and Zhang 2013: 445, fig. 3, 447, figs 5, 6). Eyes placed at the base of the ocularium (Fig. 1; see Suzuki 1974: 123, fig. 1; Schwendinger and Gruber 1992: 58, fig. 3; in comparison to Zhang and Zhang 2013: 447, fig. 6). Abdominal scutum (Fig. 1) without enlarged, dorsad-directed tubercles (see Schwendinger and Gruber 1992: 58, fig. 3 for *C. angka*).

###### Type locality.

CHINA,Yunnan Province: Zhenyuan County, Qianjiazai Town, Ailaoshan Natural Reserve, 24°16'12"N, 101°15'46"E, 2170 m, evergreen forest, extracted from leaf litter.

###### Type specimen.

Holotype male (MHBU-Opi-20160422). Adult male preserved in 75% ethanol, with genitalia in a separate microvial. Original label: MHBU-Opi-20160422, CHINA: Yunnan Province, Zhenyuan County, Qianjiazai Town, Ailaoshan Natural Reserve, 24°16'12"N, 101°15'46"E, 2170 m of elevation, 18 August 2011, A. Nakamura leg.

###### Paratype.

1♀ (MHBU-Opi-20160423), same data as the holotype.

###### Etymology.

The species epithet is a noun in apposition referring to the type locality.

###### Description of the male holotype.

Habitus as in Figs 1, 5, 19. Coloration in alcohol: dorsum yellowish brown (Fig. 19). Propeltidium with much darker brown areas. Eye rings black, hood pale tan (Fig. 21). Meso- and metapeltidium yellowish brown. Most of the opisthosomal scutum brown, only areas IV–V yellowish brown posteriorly. Venter dark brown, slightly lighter in ventral centre (Fig. 20). Chelicerae chestnut brown. Pedipalpi pale brown except for dark brown trochanters, tibiae and tarsi. Legs yellowish brown except for dark brown trochanters, metatarsi and tarsi.

**Dorsum** (Figs 5, 19). Entire body strongly sclerotized. Metapeltidium clearly separated from carapace and abdominal scutum (Figs 5, 19). Free tergites not visible from above. Surface covered with network of interconnected anvil-shaped tubercles. Anterior border of carapace with one lateral hood process on each side of ocularium. Metapeltidium with a transverse row of anvil-shaped tubercles (Figs 5, 19). Abdominal scutum with intricate lattice of interconnected anvil-shaped tubercles, its posterior margin with fence-like row of seven enlarged, posteriorly-directed digitiform tubercles. Free tergites on caudal surface of body with low keels in transverse rows (Figs 1, 20).

**Hood** (Figs 1, 5, 21) elevated above dorsal surface of body, arched, with one median, unpaired and 4 lateral, paired digitiform tubercles, diminishing in length toward base of hood; these digitiform tubercles usually with small basal cross-bars. Basal pair of digitiform tubercles connected at their base to circumocular keels touching each other above the eyes forming a short irregular median keel, the latter distally splitting into two branches.

**Venter** (Fig. 20). Coxae with dense wart-bearing setae on ventral surfaces and with dorso-distal rows of anvil-shaped tubercles; a row of anvil-shaped tubercles along anterior and posterior margins of coxae II, III and IV; coxae I and II with distal digitiform processes retro-laterally; coxa IV with similar process pro-laterally. Genital operculum short, almost tongue-shaped, surface with tubercles. Sternites with transverse rows of low keels, these reduced in the midline.

**Chelicerae** (Figs 2–4). Basal segment with a low dorso-medial tubercle, without glandular area, only ventrally and dorsally with a few setae. The basal end of second segment spherical, and with one basal pro-dorsal tooth (Fig. 2). Many long dorsal setae, and rows of short setae at base of fixed finger (Fig. 2). Fingers short, with diaphanous teeth and dark subapical teeth: one dark tooth on movable finger, two dark teeth on fixed finger (Fig. 4).

**Pedipalpi** (Figs 6–7). Trochanters with two ventral setiferous tubercles. Femora with few clavate hairs. Patellae medially with many clavate hairs and laterally with few clavate hairs. Tibiae and tarsi densely covered with clavate hairs.

**Legs.** All trochanters pro-dorsally and retro-dorsally with one enlarged tubercle. Femora, patellae and tibiae without pseudo-articulations, with distinctive microsculpture, composed of broad, thick, conical, slightly inclined denticles. Metatarsi and tarsi without annulations and microsculpture, only with setae. Tarsal segments I–II with two tarso-meres: 4 (2+2), 9 (7+2); the III–IV with three: 6 (2+2+2), 6 (2+2+2).

**Figures 13–18.**
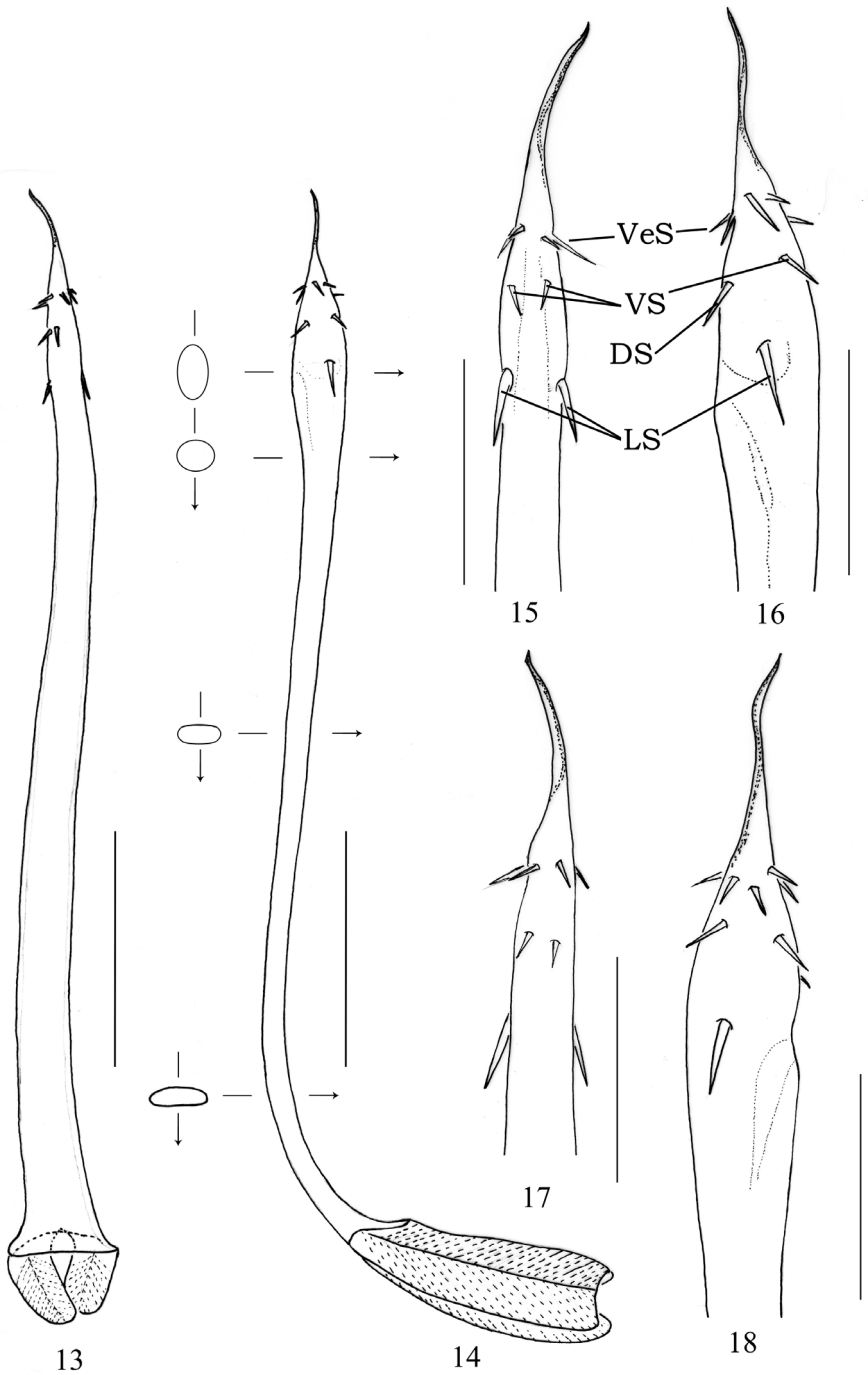
*Cladolasma ailaoshan* sp. n. male (holotype) **13** Penis, dorsal view **14** Penis, lateral view **15** Penis tip, ventral view **16, 18** Penis tip, lateral view **17** Penis tip, dorsal view. Abbreviations: **DS** dorsal spines **LS** lateral spines **VS** ventral spines **VeS** verticillate spines. Scale bars: 0.25 mm (**13–14**); 0.125 mm (**15–18**).

**Penis** (Figs 13–18) slender and lanceolate; no clear distinction between shaft, glans, and stylus. Shaft nearly parallel-sided, widened basally, then tapering distally (seen from ventral); in proximal portion dorso-ventrally depressed, in median portion elliptical and wider than long in cross-section, in distal portion close to glans almost circular in cross-section. Base of truncus dorsally bent almost at 90° together with two large lobe-like roots (seen from lateral). Glans bulged ventrally and dorsally (lateral view, Fig. 14); distal part of glans with six small spines at the base of the stylus and basal part with two small ventral and two small dorsal spines, and two large lateral spines (Figs 15–18). Stylus simple, slender, slightly torsion; tip of stylus bent.

###### Female

(Figs 8–12, 22–24). Similar in appearance and coloration to male, but the body much larger, coloration lighter (Fig. 23). Free tergites partly visible from above (Fig. 23). Hood with three lateral, paired digitiform tubercles (Figs 10, 22). Genital operculum broadly rounded, with a medial triangular flat-topped projection on anterior margin (Fig. 24). Chelicerae unarmed, only with setae (Fig. 9). Femora and Patellae of pedipalpi with many clavate hairs (Fig. 8). Tarsal segments I–IV: 3 (2+1), 9 (7+2), 7 (3+2+2), 7 (3+2+2).

**Ovipositor** (Figs 11–12). Unsegmented, short, with nonglandular setae. The apical furca with two divisions. Two *receptacula seminis* long oval saclike (Fig. 12).

###### Measurements.

Male holotype (female paratype): Total length (including hood and posterior tubercles) 2.80 (4.60). Prosoma 0.72 (0.94) long, 1.24 (1.88) wide. Opisthosoma 1.11 (1.92) long, 1.18 (2.06) wide. Median hood process 0.92 (1.19) long, 0.76 (1.13) wide. Basal segment of chelicerae 0.57 (0.77) long, 0.23 (0.32) deep; second segment of chelicerae 0.61 (0.74) long, 0.19 (0.26) deep. Penis 1.05 long (including glans), 0.10 wide at base, fork 0.26 long. Ovipositor 1.39 long. Measurements of left pedipalp and right legs as in Tables 1, 2.

**Table 1.** *Cladolasma ailaoshan* sp. n. Measurements of the pedipalp and legs of the male holotype, length/depth given for femora.

	Trochanter	Femur	Patella	Tibia	Metatarsus	Tarsus	Total
Pedipalp	0.33	0.61/0.09	0.37	0.44		0.29	2.04
Leg I	0.31	1.22/0.19	0.50	0.92	0.38	0.53	3.86
Leg II	0.35	2.68/0.16	0.88	2.37	1.53	1.33	9.14
Leg III	0.33	1.30/0.20	0.49	1.03	0.37	0.59	4.11
Leg IV	0.33	1.77/0.19	0.58	1.67	0.51	0.61	5.47

**Table 2.** *Cladolasma ailaoshan* sp. n. Measurements of the pedipalp and legs of the female paratype, length/depth given for femora.

	Trochanter	Femur	Patella	Tibia	Metatarsus	Tarsus	Total
Pedipalp	0.42	1.02/0.12	0.59	0.76		0.49	3.28
Leg I	0.40	1.89/0.29	0.82	1.46	0.56	0.55	5.68
Leg II	0.40	4.49/0.24	1.21	4.09	1.94	1.58	13.71
Leg III	0.40	2.00/0.30	0.76	1.73	0.49	0.84	6.22
Leg IV	0.48	2.75/0.30	0.83	2.86	0.66	0.94	8.52

###### Habitat.

This species was extracted from leaf litter of primeval evergreen forest using a Berlese funnel.

###### Distribution.

Known only from the type locality, the Ailaoshan National Natural Reserve in Yunnan Province, China.

###### Remarks.

After the genus *Cladolasma* was reinstated for the Asian species *C. parvulum* from Japan and *C. angka* from northern Thailand, one additional species was found, i.e., *C. damingshan* Zhang & Zhang, 2013 from subtropical southern China and in addition, the present *C. ailaoshan* sp. n., also from a subtropical environment. These specimens reinforce the distinctive characters between *Cladolasma* (Asiatic Ortholasmatinae) and *Dendrolasma* (American Ortholasmatinae) in morphological characters, e.g., metapeltidium in *Cladolasma* separated from abdominal scutum, while it is fused to it in *Dendrolasma*; *Cladolasma* with a relatively stout penis shaft, a compressed glans and a short, slender, pointed stylus, whereas *Dendrolasma* has a long, thinner shaft, a flattened glans and a contorted stylus.

According to the male genitalia of *Cladolasma* (penis unknown in *C. angka*), *C. ailaoshan* sp. n. and *C. damingshan* are clearly different from *C. parvulum*. The penial glans has a pair of large spines laterally in the new species and *C. damingshan*, while the glans has a lateral row of large spines in *C. parvulum*. Consequently, the penis of the new species shows closer relationship to *C. damingshan* than to *C. parvulum*.

The spination of glans penis follows the same pattern in the two Chinese species presently known (*C. ailaoshan* sp. n. and *C. damingshan*): the spines at the base of the stylus are arranged in a verticillate order (Figs 15–16; only small spines in *damingshan*; Zhang and Zhang 2013: 449, figs 22–24, larger ones in *C. ailaoshan* sp. n. sp.), the lateral spines are more distantly positioned from the base of stylus than in *C. damingshan*, and the two dorsal and two ventral spines are located between these two groups of spines. Additionally, the two Chinese species are different in the number of verticillate spines (six spines in *C. ailaoshan* sp. n., eight in *C. damingshan*) and by the size of the spines (small dorsal and ventral spines in *C. ailaoshan* sp. n., large ones in *C. damingshan*).

**Figures 19–24.**
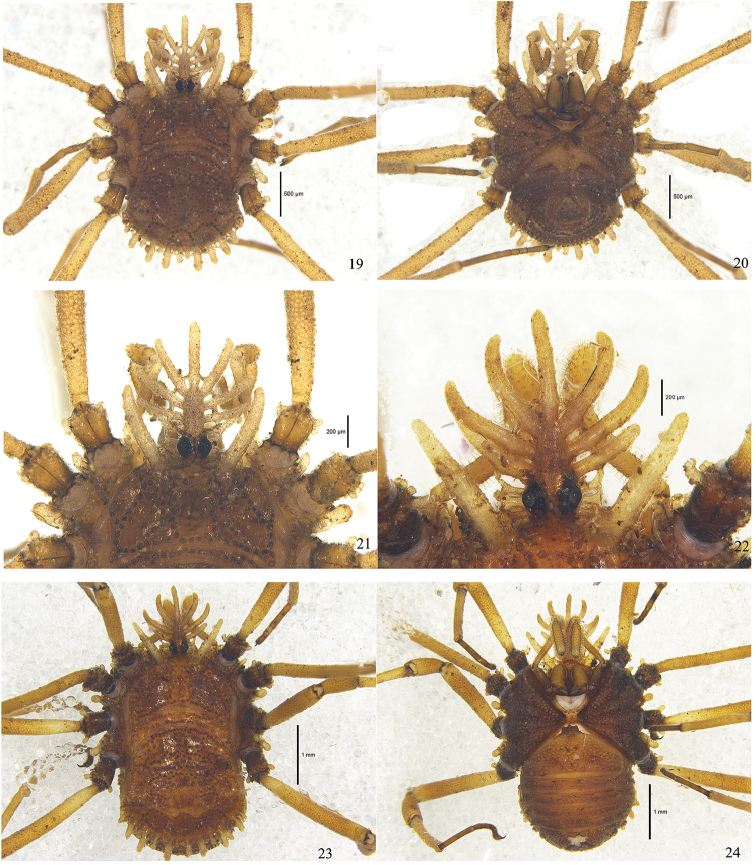
*Cladolasma ailaoshan* sp. n. Photographs of holotype male and female paratype **19** Body and parts of appendages, male, dorsal view **20** Ditto, ventral view **21** Hood, male, dorsal view **22** Hood, female, dorsal view **23** Body and parts of appendages, female, dorsal view **24** Ditto, ventral view. Scale bars: 1 mm (**23–24**); 0.5 mm (**19–20**); 0.2 mm (**21–22**).

Moreover, *C. ailaoshan* sp. n. can be easily distinguished from *C. damingshan* by the slender and curved stylus, the shape of the dorso-basal tooth on the second segment of male chelicerae, the keels around the eyes, and the location of the eyes on the hood.

## Supplementary Material

XML Treatment for Cladolasma

XML Treatment for Cladolasma ailaoshan
